# Sector Classification of Unerupted Maxillary Canines: A Deep Learning‐Based Automated Framework Using Panoramic Radiographs

**DOI:** 10.1111/ocr.70106

**Published:** 2026-03-03

**Authors:** Marzio Galdi, Davide Cannatà, Flavia Celentano, Luigia Rizzo, Domenico Rossi, Tecla Bocchino, Genoveffa Tortora, Stefano Martina

**Affiliations:** ^1^ Department of Medicine, Surgery and Dentistry University of Salerno Baronissi SA Italy; ^2^ Department of Computer Science University of Salerno Fisciano SA Italy; ^3^ Department of Neuroscience, Reproductive Science and Dentistry University of Naples Federico II Naples NA Italy

**Keywords:** artificial intelligence, deep learning, impacted maxillary canines, interceptive orthodontics, radiology

## Abstract

**Objectives:**

To develop a deep learning‐based framework to automate sector classification of unerupted maxillary canines (UMCs), assessing its accuracy and reliability compared to human ones.

**Material and Methods:**

One thousand five hundred twenty‐eight UMCs from digital panoramic radiographs (PRs) were selected using data from the Dental Department, “San Giovanni di Dio e Ruggi d'Aragona” Hospital, Salerno. After a training session with an expert in sector classification, six dental practitioners allocated UMCs of a 20% random sample of the original set (T0) in 3 different sectors according to Kim's sector classification system. The assessment was repeated after 4 weeks (T1). The accuracy and reliability of the human in determining the position of the canines were defined based on the level of agreement between the trainer and the examiners and the intra‐examiner agreement, respectively, both assessed through Cohen's K. The same radiographs were tested on different artificial intelligence (AI) models, pre‐trained on the extended dataset. The best‐performing model was identified based on its sensitivity and precision, and the model accuracy and repeatability were determined.

**Results:**

Regarding UMC allocation according to different sectors, the agreement between examiners and trainer was 0.78 (95% confidence interval = 0.77–0.80). The overall intra‐examiner agreement was 0.85 (95% confidence interval = 0.83–0.87). DenseNet121 proved to be the best‐performing model in allocating UMCs in the three different sectors, with an overall accuracy and repeatability of 76.8% and 95.3%, respectively.

**Conclusion:**

The developed framework provides an automated approach in sector classification of UMCs, whose accuracy is comparable to that of humans, but the reliability is greater.

## 1 Introduction

Maxillary canines contribute significantly to facial aesthetics and oral function, supporting the upper lip and alar base, enhancing the attractiveness of the smile through their proper size, shape, and alignment, and helping to disclose the posterior teeth during lateral jaw movements [1, 2].

Their eruption time is generally observed between 10.5 and 11.5 years of age, though this window exhibits a significant natural variation spanning three to four years [3]. With a reported prevalence of between 0.8% and 4.7%, these teeth remain impacted or erupt ectopically presumably as a result of a multitude of factors such as genetic predisposition, insufficient space within the arch, an extended eruption trajectory, and diminished dimensions or congenital absence of the adjacent lateral incisor [4, 5]. Several methods have been proposed to manage impacted or ectopic maxillary canines [6]. Among these, early diagnosis and simple interceptive procedures represent the most desirable approach, potentially streamlining or avoiding the complexity of more extensive therapy in the future, minimizing overall treatment time and cost [7, 8].

The implementation of interceptive strategies is based on various angular and linear measurements derived from radiographic imaging, typically panoramic radiographs (PRs), used to anticipate the likelihood of impaction or ectopic eruption of the canine, such as sector classification, the angle formed by the long axis of the canine and the midline, the angle formed by the long axis of the canine and the lateral incisor, the angle formed by the long axis of the canine and the occlusal plane, and the perpendicular distance from the canine cusp tip to the occlusal plane and to the midline [9, 10, 11].

Artificial intelligence (AI) methods, including deep learning (DL), are increasingly employed in dentistry for a wide range of analytical purposes, such as landmark localization, image segmentation, and object detection, progressively enabling the automation of diagnostic workflows and clinical decision‐making processes [12, 13].

A recent comprehensive review reported that current AI technologies can substantially assist dental professionals in the interpretation of PRs, achieving high accuracy in detecting carious lesions, osteoporosis, maxillary sinusitis, periodontal bone loss, periapical pathologies, and tooth identification [14]. Nevertheless, among the potential applications of AI in orthodontics, the issue of impacted canines has been largely overlooked. Indeed, although automating the classification of canine impaction on PRs could enhance clinical decision‐making and reduce both the time and effort required by practitioners, the intricate morphology of dentofacial structures and the superimposition of anatomical boundaries on PRs present considerable difficulties for AI algorithms in recognizing maxillary canine impactions [15]. Therefore, at present, no fully automated, end‐to‐end system exists for the classification and treatment planning of impacted canines.

As an initial stage in developing comprehensive fully automated tools to support clinical decision‐making pertaining to impacted maxillary canines, AI‐driven platforms that successfully detect impacted and nonimpacted maxillary canines on cropped and uncropped PRs have been demonstrated [16]. The present study aimed to advance this process by developing a deep learning‐based framework to automatically classify the position of unerupted maxillary canines (UMCs) according to the sector classification systems (SCSs) described in the literature, assessing its accuracy and reliability compared to manual assessment.

## 2 Methods

### 2.1 Ethical Approval and Consent

The present research protocol was developed following the STARD (Standards for Reporting of Diagnostic Accuracy Studies) and CLAIM (Checklist for Artificial Intelligence in Medical Imaging) checklists [17, 18]. The study received approval from the Campania Region 2 Ethics committee (protocol code: 2024/30960). Written informed consent was obtained from all patients (or their parents/guardians if a minor) at the time of the initial screening visit at the Dental Department of the Hospital “San Giovanni di Dio e Ruggi d'Aragona” in Salerno. The written consent indicated that data and radiographs would be used for research and education purposes without identification. After receiving ethical approval on December 6, 2024, the Dental Unit obtained access to data collection from the medical reports and radiographs of the patients. Data access for the retrospective collection of information commenced on December 20, 2024.

### 2.2 Study Sample and Dataset

A retrospective analysis of 17,854 digital PRs was conducted using data from the Dental Department of the Hospital “San Giovanni di Dio e Ruggi d'Aragona” in Salerno, taken between 2013 and 2024. PRs were screened by two dentists (LR and SM) according to the following criteria:

Inclusion criteria:

- high quality PRs with good visibility of anatomic structures;
- presence of unilateral (either right or left) or bilateral UMCs;
- patients older than 9 years, as it is around the age of 9 that the canines, as part of their eruption process, reach their maximum mesial inclination before curving distally to erupt in the arch [19];
- no gender restrictions

Exclusion criteria:

- Poor‐quality PRs or PRs with reduced visibility of anatomic structures:
- pathological conditions (endocrine deficiencies such as hypothyroidism, hypopituitarism, trauma, or jaw fractures), and hereditary diseases or syndromes such as Down syndrome and cleidocranial dysostosis affecting facial or dental growth patterns;
- absence of lateral incisors adjacent to the UMC;
- dental anomalies of the upper incisors:
- presence of a neoformation (cyst, odontoma) in the upper jaw.

When PRs displayed bilateral UMCs, they were split into two radiographs (A—right side and B—left side) and considered separately in both training and validation set.

### 2.3 Classifications

**FIGURE 1:**
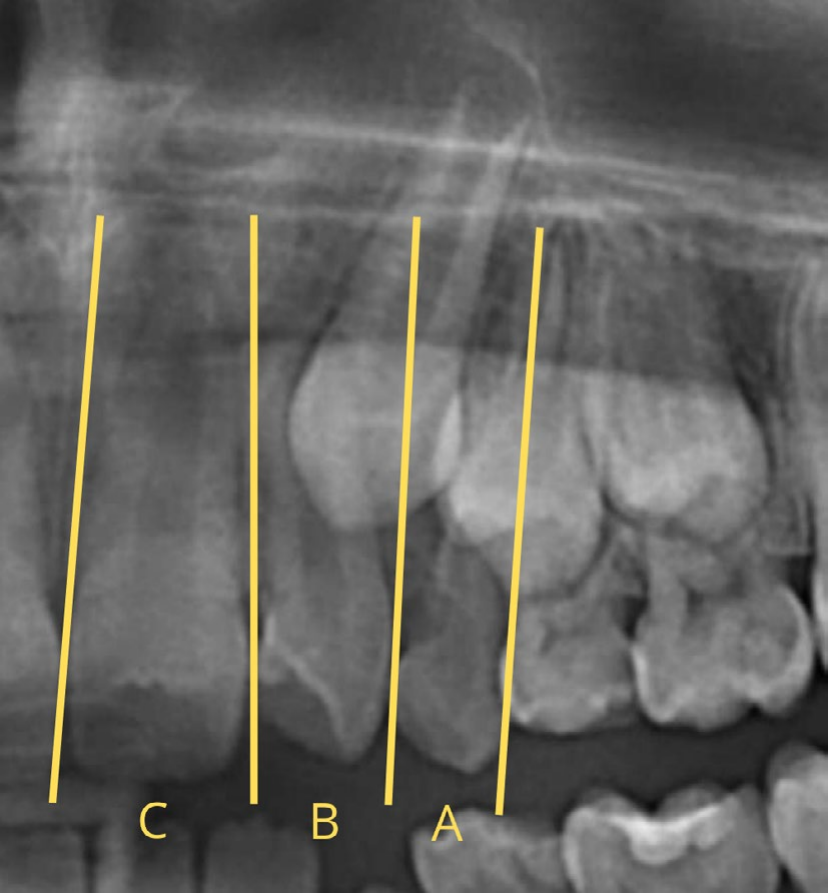
3‐sector classification system proposed by Kim et al. Sector A – the area distal to a line tangent to the distal heights of the contour of the lateral incisor crown and root; Sector B – the area between a line tangent to the distal heights of the contour of the lateral incisor crown and root and a line tangent to the mesial heights of the contour of the lateral incisor crown and root; Sector C – the area mesial to a line tangent to the mesial heights of the contour of the lateral incisor crown and root.

An orthodontist expert in sector classification, i.e., an orthodontist with over 10 years of clinical experience (SM), determined the position of UMCs in the selected PRs according to three different sector classification systems proposed in the literature, namely the 5– [9, 10], 4– [20] and 3– [21] sector classification systems. Since the 3‐sector classification system allowed the sample to be divided into numerically homogeneous groups, it was selected for examiner training and the development of DL model. Figure 1 illustrates the 3‐ sector classification system [21] (Figure 1).

### 2.4 Examiners and Calibration

Six practitioners randomly selected from the group of clinicians of the Department of Medicine, Surgery, and Dentistry “Scuola Medica Salernitana”, University of Salerno, Italy, were asked to classify UMCs featured in PRs according to the 3‐sector classification system. Before the assessment, all the examiners attended a one‐hour training session, during which the expert in sector classification (SM) trained them in identifying sectors and determining the position of canines on a set of PRs with UMCs.

After the training session, a random sample of 20% of UMCs from PRs taken from the original set was provided to the examiners, who were invited to locate the medial crown position of the UMC in one of the three sectors described by Kim [21]. Each examiner was invited to perform the sector classification in a quiet room, without time limit (time T0). After a 4‐week interval, all the examiners were invited to repeat the sector classification with the same digital radiographs placed in a different order (time T1).

### 2.5 Statistical Analysis

The accuracy of the human examiners in determining the position of the canines was defined based on the level of agreement between the trainer (SM) and the examiners, at the different observations (T0 and T1), assessed through Cohen's K. The range of variation of the K statistic is between 0 for no agreement and 1 for perfect agreement with five intermediate levels: *‘slight agreement’* (0.01–0.20), ‘*fair agreement’* (0.21–0.40), ‘*moderate agreement’* (0.41–0.60), ‘*substantial agreement’* (0.61–0.80) and ‘almost perfect agreement*’* (0.81–0.99) [22]. The weighted mean of the Cohen's K coefficients was calculated using a fixed‐effects meta‐analysis, with weights based on the variance derived from the confidence intervals.

The reliability of the human examiners in determining the position of the canines was evaluated based on the intra‐examiner agreement between the two observations (T0 vs. T1).

To estimate Cohen's kappa, a sample size of 96 subjects was needed, based on a 95% confidence interval (CI), a margin of error of 0.2, an expected positive test rate of approximately 20%, and without making any assumptions about the true kappa value.

### 2.6 DL Model Development

#### 2.6.1 Image Preprocessing and Data Encoding

The deep learning pipeline was developed to address the task of sector classification of unerupted maxillary canines on panoramic radiographs. The model operates on images in which the presence of an unerupted canine had been previously identified through expert assessment; accordingly, no automated detection, localization, or segmentation modules were included in the pipeline. Panoramic radiographs were manually selected to ensure the presence of unerupted maxillary canines, and the network was trained exclusively to assign the canine to one of the predefined anatomical sectors defined by the selected classification system. As such, the proposed approach focuses on classification rather than on end‐to‐end automation of the diagnostic workflow.

All panoramic radiographs were resized to a uniform spatial resolution of 224 × 224 pixels to ensure compatibility with the convolutional backbones employed. Images were normalised using channel‐wise mean and standard deviation normalisation, consistent with ImageNet‐pretrained networks. Prior to resizing, a manual region‐of‐interest (ROI) cropping was performed to focus the input on the maxillary region containing the unerupted canine, based on expert assessment. No automated histogram equalisation or denoising filters were applied, in order to preserve the original radiographic characteristics and avoid introducing artificial image alterations.

For supervised learning, sector labels were encoded using one‐hot encoding, enabling multi‐class classification across the three anatomical sectors. Clinical data were prepared separately and included as complementary input for the multimodal teacher model.

To improve model robustness and reduce overfitting, on‐the‐fly data augmentation was applied during training. Augmentation operations included random rotations, translations, horizontal flipping, zooming, and brightness/contrast variations. These transformations were applied within conservative ranges to simulate plausible variations in patient positioning and radiographic acquisition while preserving anatomical consistency. No data augmentation was applied to the validation set.

#### 2.6.2 Dataset Split and Class Imbalance Handling

The dataset consisted of 1528 unerupted maxillary canines derived from 1066 panoramic radiographs. A stratified train–validation split (80%/20%) was implemented using StratifiedShuffleSplit, ensuring balanced class representation across subsets.

Class imbalance was addressed during training by computing class weights based on the inverse frequency of each sector, which were incorporated into the loss function. No oversampling or undersampling strategies were applied.

#### 2.6.3 Model Architecture

To improve computational efficiency while preserving predictive performance, a knowledge distillation framework was adopted to train the proposed deep learning model. This approach relies on transferring knowledge from a larger and more expressive teacher network to a smaller and more lightweight student model, enabling effective deployment in clinical settings while maintaining high classification accuracy.

The teacher model was designed as a multimodal architecture integrating panoramic radiographs and structured clinical metadata, including canine depth, beta angle, and root maturity. Radiographic features were extracted using a DenseNet121, a network architecture selected for its proven efficacy in medical imaging. This backbone was pre‐trained on ImageNet and subsequently fine‐tuned to adapt to the dental imaging domain. The extracted image features were merged with the processed clinical metadata to generate the final sector classification. The teacher model was trained using standard optimization algorithms and regularization strategies to ensure training stability and prevent overfitting.

Once trained, the teacher's predicted probability distributions were utilised to guide the student model. Crucially, the student network replicated only the image‐processing pathway, excluding clinical metadata. Training was driven by a composite loss function that encouraged the student to mimic the teacher's outputs. Through this process, the student model successfully approximated the predictive behaviour of the multimodal teacher while relying exclusively on radiographic input, resulting in a compact architecture suitable for routine clinical application when structured patient data are unavailable.

#### 2.6.4 Model Selection

A preliminary evaluation phase was conducted to compare multiple convolutional neural network (CNN) architectures as potential teacher models for the classification of anatomical sectors in PRs. The goal was to identify models capable of capturing the spatial and structural complexity of dental anatomy in 2D imaging. The selection process focused on architectural design, training behavior, and theoretical suitability for medical image classification tasks. The following CNN models were included in the comparison: ResNet50 [23], EfficientNetV2 [24], EfficientNetB0 [25], DenseNet121 [26].

#### 2.6.5 Training Protocol

Both teacher and student models were trained on the same stratified training set (80%) and evaluated on the same validation set (20%) to ensure comparability. Teacher predictions used for distillation were computed exclusively on the training subset. No test set external to the validation cohort was employed.

#### 2.6.6 Model Performance Evaluation

The performance of the classification models was assessed using a comprehensive set of evaluation metrics widely adopted in machine learning. Core classification metrics included accuracy, precision, and recall, with particular emphasis on weighted recall to account for class imbalance across the sector categories.

In addition, although the task was formulated as a multi‐class classification problem, the three anatomical sectors were treated as ordered categories reflecting a progressive mesial displacement of the unerupted canine. Under this ordinal assumption, regression‐based metrics were also computed as complementary indicators of prediction error magnitude.

To further quantify the discrepancy between predicted and actual class labels, the sector classes were encoded as ordinal values, and regression‐based metrics were also computed, including Mean Absolute Error (MAE), Mean Squared Error (MSE), and Root Mean Squared Error (RMSE). Formal definitions and formulas for all evaluation metrics are reported in Data S1.

All model performance metrics were computed using the sector assignments provided by the expert orthodontist (SM) as the reference standard (ground truth). This expert annotation served as the sole comparator for evaluating model outputs; no panel‐based consensus procedure was employed.

#### 2.6.7 Software and Hardware Environment

Model development and training were conducted using TensorFlow (Keras API). Training was performed on a workstation equipped with a GPU accelerator, under a standard Linux‐based operating system. All experiments were executed in a controlled software environment to ensure consistency across runs.

## 3 Results

### 3.1 Sample Selection Process

Of the 17,854 PRs that were analysed, a total of 1066 were included in the final dataset, yielding a total of 1528 UMCs, according to the inclusion and exclusion criteria defined in the study protocol. Therefore, the present study sample consisted of 1528 UMCs. Based on the trainer analysis, the distribution of the samples across the three anatomical sectors was as follows: 592 samples were assigned to Sector A; 568 samples to Sector B; 368 samples to Sector C.

### 3.2 Analysis of Human Accuracy and Reliability in Sector Classification

The results from the training evaluation found an agreement between the examiners and the trainer ranging from substantial to almost perfect (Table 1), with an overall agreement (Cohen's K) of 0.78 (95% confidence interval = 0.77–0.80).

**TABLE 1:** Training evaluation process: Agreement (Cohen's K) between trainer and examiner in sector classification according to the 3‐sector classification system.

Examiner	T0 K value (95% CI)	T1 K value (95% CI)
1	0.81 (0.76–0.87)	0.75 (0.69–0.82)
2	0.87 (0.81–0.91)	0.86 (0.81–0.91)
3	0.79 (0.73–0.84)	0.74 (0.68–0.81)
4	0.69 (0.62–0.76)	0.63 (0.56–0.70)
5	0.73 (0.66–0.80)	0.69 (0.62–0.76)
6	0.82 (0.76–0.87)	0.82 (0.77–0.88)
Overall	0.78 (0.77–0.80)	

Abbreviations: CI, confidence interval; T0, time of examiners' first analysis of radiograph; T1, time of second examiners ‘analysis of radiographs.

The agreement between observations (T0 versus T1) for each examiner ranged from 0.80 to 0.92 (Table 2), with an overall intra‐examiner agreement (Cohen's K) of 0.85 (95% confidence interval = 0.83–0.87).

**TABLE 2:** Intra‐observer (T0 versus T1) agreement for each examiner assessed through Cohen's K.

Examiner	K value (95% CI)
1	0.81 (0.75–0.86)
2	0.87 (0.83–0.92)
3	0.81 (0.75–0.87)
4	0.90 (0.86–0.94)
5	0.80 (0.74–0.86)
6	0.92 (0.88–0.96)
Overall	0.85 (0.83–0.87)

Abbreviations: CI, confidence interval; T0, time of examiners' first analysis of radiograph; T1, time of second examiners' analysis of radiographs.

### 3.3 Development of DL‐ Based Framework for Sector Classification

#### 3.3.1 Dataset Composition

The dataset that was used for DL model development consisted of 1528 UMCs, classified according to the 3‐sector classification system.

#### 3.3.2 CNN Model Selection

Among the evaluated CNN architectures, DenseNet121 achieved the highest overall accuracy of 76.4% in sector classification. In comparison, ResNet50 reached an accuracy of 67.2%, EfficientNetV2 achieved 58.4%, and EfficientNetB0 obtained 49.6%. Given its superior performance, DenseNet121 was chosen as the teacher model for knowledge distillation.

#### 3.3.3 Model Training

A two‐step strategy based on DenseNet121 was adopted. First, a multimodal *teacher model* was trained using both PRs and structured clinical data (canine depth, angular deviation, and root maturity). The image branch of the network used a DenseNet121 backbone pretrained on ImageNet, fine‐tuned on our dataset. Then, a student model was trained using only radiographs, replicating the teacher's predictions via knowledge distillation (cross‐entropy + divergence loss).

**FIGURE 2:**
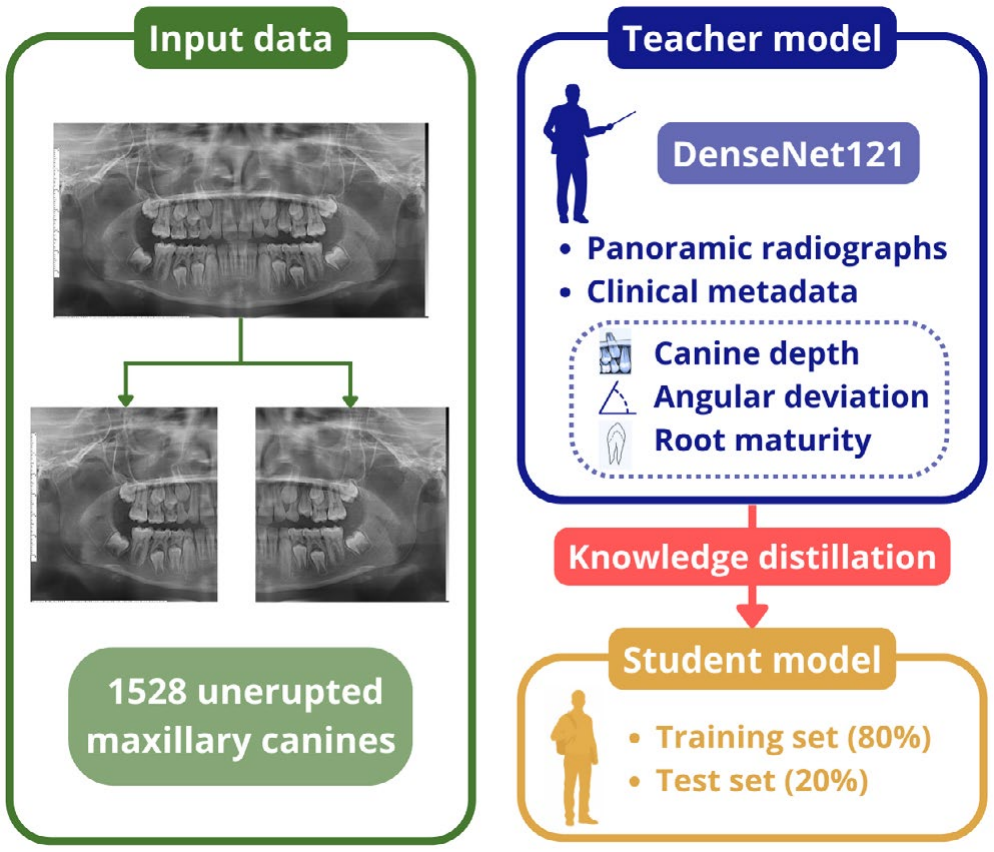
Workflow of the AI model development using panoramic radiographs for unerupted maxillary canine detection, employing a knowledge distillation approach with DenseNet121.

Both models were trained on the same dataset, consisting of 1222 images (80%) for training and 306 images (20%) for validation, ensuring consistency in performance comparison (Figure 2).

#### 3.3.4 Classification Results

The model was evaluated on a validation set of 306 UMCs from PRs (20% of the full dataset). The student model, trained via knowledge distillation and relying exclusively on image input, achieved an overall classification accuracy of 76.47%. A per‐class analysis revealed marked differences in sensitivity and precision across the three sectors.

- Sector A was correctly identified in 114 out of 118 cases (recall 96.61%, precision 71.25%), indicating high sensitivity but an overprediction tendency, likely due to class imbalance and visual similarity with adjacent sectors.
- Sector B showed high precision (87.50%), whereas recall fell to 49.56%, with many cases misclassified as Sector A, reflecting the anatomical ambiguity of this intermediate region.
- Sector C exhibited a precision of 76.19% and a recall of 85.33%, correctly classifying 64 out of 75 images.

A more detailed analysis of prediction errors is provided by the confusion matrix, which maps true class labels against predicted outputs. As shown in Table 3, Sector C showed the highest consistency, while Sector B was most frequently misclassified as Sector A.

**TABLE 3:** Confusion matrix comparing AI model predictions against the expert trainer's gold standard for UMC sector classification.

Canine's sector according to the trainer	Number of canines classified by the model in sector A	Number of canines classified by the model in sector B	Number of canines classified by the model in sector C
Sector A	114	2	2
Sector B	41	56	16
Sector C	5	6	64

#### 3.3.5 Performance Metrics

Model performance was assessed through standard quantitative metrics. The Mean Absolute Error (0.258) and Mean Squared Error (0.304) indicated low deviation and infrequent severe errors, while the Root Mean Squared Error (0.551) confirmed close alignment between predictions and true labels. The model achieved an overall accuracy of 76.47% and a recall of 77.17%, demonstrating robust and sensitive classification performance based solely on radiographic input. When subjected to repeated tests, the model showed a repeatability of 95.3%.

## 4 Discussion

The present study developed and validated a DL model to automate the sector classification of UMCs on digital PRs, comparing its accuracy and reliability with those of human operators. The results indicated that the DenseNet121 architecture, trained using a knowledge distillation strategy, can classify the position of canines with an overall accuracy of 76.8% and a repeatability of 95.3%, suggesting it is a promising tool for clinical decision support.

A first significant finding that emerged from the present analysis concerns the performance of human examiners. The agreement between examiners and the expert trainer was found to be “substantial” (Cohen's K = 0.78), while intra‐examiner agreement was “almost perfect” (Cohen's K = 0.85). These values confirm that sector classification on two‐dimensional radiographs, although standardised by Kim's method, remains subject to some interpretative variability, probably due to the intrinsic complexity of anatomical overlaps in PRs [27].

In this context, the developed AI model showed accuracy comparable to that of humans (76.8%), but with significantly higher repeatability (95.3%). Similar results have been found in previous studies comparing DL models with dentists in assessing bone quality on CBCT images according to the Lekholm and Zarb classification [28]. The capability of DL models to outperform human repeatability may be explained by the fact that while human operators are subject to fatigue, distractions, or cognitive biases that can influence diagnosis over time, the algorithm offers almost absolute consistency of judgment [29]. This suggests that AI should not necessarily replace the clinician but serve as a standardised “second opinion” to reduce diagnostic variability, especially in large‐scale screening contexts [30].

Among the architectures tested, DenseNet121 outperformed ResNet50 and EfficientNet, proving to be the most suitable for capturing the complex anatomical features of PRs. Consistently, DenseNet‐121 has proved to be highly effective for medical imaging applications because of its high level of connectivity that makes it efficient in image detection and classification tasks. Notably, in dental radiology, Lee et al. demonstrated that DenseNet‐121 could classify dental caries with a level of accuracy comparable to that of expert dentists, achieving 95.7% accuracy in the detection of early dentinal lesions [31]. Moreover, DenseNet‐121 achieved outstanding accuracy of 99.5% in classifying common dental pathologies such as periapical periodontitis, caries, and cysts on digital dental PRs, surpassing other CNN models [32]. The use of knowledge distillation made it possible to obtain a lightweight “student model” based solely on images, which, however, benefited during training from clinical information (depth, angle) processed by the “teacher model.” This approach makes the model easy to implement in everyday clinical practice, where structured clinical metadata may not always be available [33].

An analysis of performance by sector reveals some interesting considerations. The model showed excellent sensitivity for Sector A (96.61%), but a tendency to overestimate (accuracy 71.25%). In contrast, Sector B represented the greatest challenge, with a high rate of misclassification towards Sector A. This difficulty is intrinsic to the nature of panoramic radiography: being a 2D representation of a 3D structure, the transition zone between sectors (particularly the overlap with the lateral incisor) is often subject to geometric distortions and blurring. It is plausible that the model, faced with the visual ambiguity of Sector B, tends to converge towards the most represented or visually similar class (Sector A) [1]. From a clinical perspective this misclassification is relevant as the canines in Sector B generally represent the cases with the highest risk of impaction and root resorption of the lateral incisor, which require interceptive treatment at an early age (i.e., extraction of the primary canine or orthodontic traction), whereas the canines in Sector A usually have a more favourable eruption pattern and can be considered for observation only [34, 35]. Consequently, an error in the model that overestimates Sector B might lead to unnecessary treatment, whereas the underestimation of Sector A might delay the appropriate treatment; thus, the need for a correct and reliable classification is evident. This may mean that enhanced model attention mechanisms or the inclusion of auxiliary clinical features beyond image input are needed.

Sector C, on the other hand, showed a good balance between precision and recall, suggesting that when the canine is in an extreme mesial position, the model is able to distinguish it more clearly. Since sector C represents an unfavourable prognostic factor for interceptive approaches, the sensitivity of the model for canines positioned in this sector allows the clinician to be directed towards second‐level instrumental examinations that are generally necessary to plan surgery.

Early diagnosis of canine impaction is crucial to plan interceptive interventions [36]. The literature suggests that the use of AI in orthodontics has focused mainly on tooth detection or automated cephalometry [14]. The present study takes a step forward, moving from simple detection to positional classification, which is a fundamental prerequisite for prognosis and treatment selection. The integration of this tool into radiographic visualisation software could act as an automatic alert system, signalling the probable severity of the impaction to general dental practitioners and suggesting the need to refer the management to a specialist.

It is important to note that the intent of Ericson and Kurol's positional classification of UMCs and subsequent revisions was to provide a prognostic indicator for interceptive orthodontic interventions. Therefore, the usefulness of this model is strictly limited to interceptive approaches and does not extend to the planning of surgical interventions for the recovery of impacted canines, for which second‐level instrumental examinations are necessary. It should also be noted that this tool cannot replace the clinician in diagnosis and treatment decisions, which cannot be based solely on radiographic examinations but must consider other aspects that can only be identified through a detailed clinical examination.

This study has some limitations. First, the exclusive use of PRs; although PR is the standard first‐level examination, it suffers from distortions and magnifications that do not allow vestibular/palatal localization of the canine, a critical factor for surgical planning. However, the aim of the model is screening and initial classification, not definitive 3D surgical planning. Secondly, the proposed deep learning pipeline was designed to perform sector classification under the assumption that the presence of an unerupted maxillary canine had already been identified on the panoramic radiograph. Accordingly, no automated detection, localization, or segmentation step was implemented, and manual preselection of radiographs was required prior to model application. As a consequence, the present approach focuses on sector classification and cannot be considered a fully end‐to‐end automated diagnostic system.

Moreover, the imbalance of classes (with the prevalence of sectors A and B over C) may have influenced the training, despite the use of weighted metrics. The choice of the 3‐sector system to limit the imbalance of classes represents another major issue.

Finally, the study is retrospective and carried out in a single center with a single expert trainer and with a relatively homogeneous patient population and radiographic acquisition setting; external validation on datasets from different centers and radiographic equipment will be necessary to increase the model's robustness and generalizability.

Future developments should therefore focus on expanding the dataset, particularly regarding the underrepresented sectors, in order to strengthen the model's ability to distinguish between all sectors. Increasing the number and type of samples with X‐rays showing agenesis and cysts would mitigate class imbalance, improve robustness, and reduce overfitting towards dominant patterns.

## 5 Conclusion

The developed deep learning‐based framework offers an automated approach to the sector classification of UMCs with accuracy comparable to that of human examiners and consistent reliability. While challenges persist in discriminating cases within intermediate sectors (Sector B) due to the inherent limitations of 2D imaging, this tool may contribute to the automation of orthodontic diagnostic workflows and could potentially assist clinicians in the early detection of eruption anomalies.

## Author Contributions

Conceptualization, G.T. and S.M.; methodology, M.G. and D.C.; software, L.R., F.C. and D.R.; validation, S.M., G.T. and M.G.; formal analysis, M.G. and D.C.; investigation, F.C.; resources, T.B.; data curation, M.G., D.C. and L.R.; writing – original draft preparation, D.C. and M.G.; writing – review and editing, S.M. and G.T.; visualisation, G.T.; supervision, S.M.

## Funding

The authors have nothing to report.

## Ethics Statement

The study received approval from the Campania Region 2 Ethics committee (protocol code: 2024/30960).

## Consent

All the participants gave their consent to participate anonymously to the study.

## Conflicts of Interest

The authors declare no conflicts of interest.

## Supporting information


**Data S1:** Appendix.

## Data Availability

The data that support the findings of this study are available on request from the corresponding author. The data are not publicly available due to privacy or ethical restrictions.
