# Supplementary material for: Sector Classification of Unerupted Maxillary Canines: A Deep Learning‐Based Automated Framework Using Panoramic Radiographs
Source: Orthod Craniofac Res. 2026 Mar 3;29(4):631–9. doi: 10.1111/ocr.70106 (PMC13485220; doi:10.1111/ocr.70106)
Supplement: Supplementary file 1 [file OCR-29-631-s001.docx]

# **APPENDIX**

**Evaluation Protocol**

Model performance was evaluated using standard metrics derived from classification outcomes (true positives, false positives, true negatives, false negatives). The following metrics were computed:

- **Accuracy (ACC)**
  Proportion of correct predictions over the total cases:

- **Recall (Sensitivity)**
  Ability to correctly identify all true cases of each class:

A weighted version was calculated to account for class imbalance:

- **Precision**
  Proportion of correct positive predictions:

- **Mean Absolute Error (MAE)**
  Average absolute difference between prediction and reality:

- **Mean Squared Error (MSE)** and **Root Mean Squared Error (RMSE)**
  Indicators of overall prediction error and its magnitude:

These metrics provided a comprehensive picture of how well the models performed, both in terms of classification accuracy and prediction reliability.
